# Supplementary material for: All-at-once RNA folding with 3D motif prediction framed by evolutionary information
Source: Nat Methods. 2025 Oct 3;22(10):2094–106. doi: 10.1038/s41592-025-02833-w (PMC12510878; doi:10.1038/s41592-025-02833-w)
Supplement: Supplementary file 3 — Source code to run CaCoFold-R3D. Data and results. [file 41592_2025_2833_MOESM3_ESM.gz › supplemental_material/results/CaCoFold-R3D/CaCoFold-R3D_Rfam_vshuffle/RF01871_MALAT1.R2R.sto.pdf]

RF01871_MALAT1

5′ - U A A Y G A A U U G A U G A G A A ● U R C A A U G A A G A ● C ● A A U G U C C A U C U C A A A A U A C U G C U U U U A C A A A A G C A G A A U R ● A A R ● G A A R ● R A A A U G A A A A U ● ○
